# HIV Latency-Reversing Agents Have Diverse Effects on Natural Killer Cell Function

**DOI:** 10.3389/fimmu.2016.00356

**Published:** 2016-09-21

**Authors:** Carolina Garrido, Adam M. Spivak, Natalia Soriano-Sarabia, Mary Ann Checkley, Edward Barker, Jonathan Karn, Vicente Planelles, David M. Margolis

**Affiliations:** ^1^UNC HIV Cure Center, University of North Carolina at Chapel Hill, Chapel Hill, NC, USA; ^2^Department of Medicine, University of North Carolina at Chapel Hill, Chapel Hill, NC, USA; ^3^Department of Medicine, University of Utah School of Medicine, Salt Lake City, UT, USA; ^4^Department of Molecular Biology and Microbiology, School of Medicine, Case Western Reserve University, Cleveland, OH, USA; ^5^Department of Immunology and Microbiology, Rush University Medical Center, Chicago, IL, USA; ^6^Department of Pathology, University of Utah School of Medicine, Salt Lake City, UT, USA; ^7^Department of Microbiology and Immunology, University of North Carolina at Chapel Hill, Chapel Hill, NC, USA

**Keywords:** HIV, natural killer cells, latency reversing agents, immune function

## Abstract

In an effort to clear persistent HIV infection and achieve a durable therapy-free remission of HIV disease, extensive pre-clinical studies and early pilot clinical trials are underway to develop and test agents that can reverse latent HIV infection and present viral antigen to the immune system for clearance. It is, therefore, critical to understand the impact of latency-reversing agents (LRAs) on the function of immune effectors needed to clear infected cells. We assessed the impact of LRAs on the function of natural killer (NK) cells, the main effector cells of the innate immune system. We studied the effects of three histone deacetylase inhibitors [SAHA or vorinostat (VOR), romidepsin, and panobinostat (PNB)] and two protein kinase C agonists [prostratin (PROST) and ingenol] on the antiviral activity, cytotoxicity, cytokine secretion, phenotype, and viability of primary NK cells. We found that *ex vivo* exposure to VOR had minimal impact on all parameters assessed, while PNB caused a decrease in NK cell viability, antiviral activity, and cytotoxicity. PROST caused non-specific NK cell activation and, interestingly, improved antiviral activity. Overall, we found that LRAs can alter the function and fate of NK cells, and these effects must be carefully considered as strategies are developed to clear persistent HIV infection.

## Introduction

The recent description of an HIV-1 infected individual who experienced a sterilizing cure (1), without evidence of replication-competent virus *in vivo*, and others in whom early antiretroviral therapy (ART) resulted in undetectable viremia and maintenance of immune competence despite the cessation of ART (a functional cure) (2), has given rise to a variety of experimental approaches to induce cure or drug-free remission of HIV-1 infection. The most intensively studied eradication strategy [known as “shock and kill” (3)] rests on inducing viral expression within latently infected CD4^+^ T cells, with the goal of reducing the reservoir size through viral cytopathic effects (CPE) or immune-mediated clearance. However, recent *in vitro* experiments demonstrated that proviral reactivation alone did not result in viral CPE, and the autologous HIV-1 specific CD8^+^ T cells of patients were unable to clear reactivated cells (4). Clearly, the capacity of the host immune system to recognize and kill infected cells upon reactivation requires closer evaluation.

Histone deacetylase (HDAC) inhibitors and protein kinase C (PKC) agonists are two promising classes of latency-reversing agents (LRAs) that are undergoing extensive testing in *in vitro* models and in initial pilot clinical trials to reactivate latent HIV-1 infection. HDAC inhibitors were developed as anticancer drugs as HDACs play important roles in epigenetic and non-epigenetic transcriptional regulation, inducing apoptosis and cell cycle arrest (5). In the context of HIV-1 reactivation, HDAC inhibitors induce transcription at the HIV-1 long terminal repeat (LTR) (6–9). PKC agonists induce latent viral expression though NF-κB signaling (10). Members of these two LRA classes have demonstrated efficacy in inducing HIV-1 expression in cells from patients on ART *in vivo* and *in vitro* (9, 11–16). However, as both histone deacetylation and signaling through NF-κB may impact the function of diverse cell populations, the effect of LRAs beyond latently infected cells must be carefully evaluated.

The influence of LRAs on cytotoxic T-lymphocytes (CTL) has recently been assessed. In one *in vitro* study, selected HDAC inhibitors caused a negative impact on CTL effector function (17), although in both this study and in another study that focused on vorinostat (VOR) (18), little effect of a pharmacologically relevant exposure to VOR was seen. CD8^+^ T cells are a well-studied and crucial effector cell population contributing to target cell clearance after viral reactivation. However, other effector subsets may also play an important role, including cells from the innate immune system. Natural killer (NK) cells are the main effectors of the innate immune response. NK effector function is elicited immediately upon recognition of activating ligands without prior exposure to the infected cell or to viral antigens, resulting in direct lysis of target cells and/or promotion of antibody-dependent cellular cytotoxicity (ADCC) (19). In addition, NK activity has been associated with HIV post-treatment control of viremia after treatment interruption (20), ADCC has been correlated with protection in a recent HIV-1 vaccine trial (21) and innate immune cell responses were correlated with HIV-1 DNA decline during panobinostat (PNB) treatment *in vivo* (22). Thus, multiple lines of evidence suggest the relevance of NK cells in the clearance of persistent HIV-1 infection.

In the present study, we aim to better understand the impact of LRAs on the innate immune system, and specifically on NK cells. LRAs might impact the capacity of NK cell to clear infected cells in at least two ways: (i) through a direct impact on immune effector cells, causing activation, toxicity, or modifying receptor expression and cytotoxicity capacity or (ii) affecting the expression of ligands in the target population modifying effector recognition and subsequent clearance. Herein, we analyze both the direct impact of candidate compounds from two promising LRA classes on NK cells, and the effects on ligand expression on target cells *ex vivo*, as a means of informing HIV-1 eradication strategies making use of these agents in future pilot clinical trials.

## Materials and Methods

### Cell Samples

Peripheral blood mononuclear cells (PBMC) were obtained by Ficoll gradient from buffy coats of HIV-1 negative healthy donors, under approval of the UNC Biomedical Institutional Review Board. NK and CD4^+^ T cells were magnetically isolated from the PBMCs by negative selection (StemCell Technologies, Vancouver, BC, Canada). The NK cell enrichment antibody cocktail included monoclonal antibodies against CD3, CD4, CD14, CD19, CD20, CD36, CD66b, CD123, HLA-DR, and glycophorin A. The CD4^+^ T cell enrichment antibody cocktail included specific antibodies against CD8, CD14, CD16, CD19, CD20, CD36, CD56, CD66b, CD123, TCR-γ/δ, and glycophorin A. After isolation, NK cells were cultured in Iscove’s Modified Dulbecco’s Medium (IMDM) supplemented with 10% heat inactivated bovine serum and 5% penicillin plus streptomycin (cIMDM), including or not the different LRAs at the appropriate concentrations for 24 h. Then, cells were washed and functional assays performed.

### Ethics Statement

Study participants provided written informed consent under a protocol that was approved by the UNC Biomedical Institutional Review Board.

### Latency-Reversing Agents

The compounds comprising our LRA panel were provided, and stocks prepared, by the CARE Pharmacology Core of the University of North Carolina. VOR was donated by Merck, and romidepsin (RMD) and PNB obtained from Selleckhem. For all three, a 10 mM stock was prepared in DMSO, and further diluted with IMDM to a working stock concentration of 25 μM (VOR) or 5 μM (RMD and PNB). Prostratin (PROST) was purchased from Cayman Chemical (Ann Arbor, MI, USA) in an ethanol solution, which was lyophilized and reconstituted with DMSO to a concentration of 5 mM, and further diluted in plain IMDM to a working stock solution of 25 μM. Ingenol (ING) 3-20 dibenzoate was obtained from Santa Cruz Biotechnology; stock solution was prepared in DMSO at a concentration of 1 mM and diluted to working stock concentration of 25 μM with non-supplemented IMDM. All stocks and working solutions were stored at −20°C and used avoiding repeated freezing–thawing cycles. LRA concentrations used in the experiments were selected based on pre-clinical data to reflect the potential physiological concentration (23–25). VOR was used at a concentration of 335 nM, RMD at 10 nM, PNB at 20 nM, PROST at 1000 nM, and ING at 100 nM. If physiologic (*in vivo*) data were not available for selecting a concentration, concentrations were chosen to reflect dosing previously shown to be effective in HIV-1 reactivation in *ex vivo* studies (11, 26, 27). In addition, a lower and a higher dose of the one considered physiological were tested in some experiments to determine if there was a dose-dependent relationship.

### Viral Inhibition Assays

CD4^+^ T cells were isolated by negative selection in parallel to NK cells from each donor. Isolated CD4^+^ T cells were activated during 24 h with 2 μg/mL PHA (Sigma Aldrich, St Louis, MO, USA) and 60 U/mL IL-2 (Peprotech, Rocky Hill, CT, USA). Cells were then infected with the JR-CSF viral strain by spinoculation for 90 min at 2500 rpm. After spinoculation, cells were extensively washed to remove free virions and 50,000 CD4^+^ T cells were plated in triplicate for each condition in a 96-well plate. NK cells, previously exposed to LRAs or not (reference control), were added to the wells in an effector:target (E:T) ratio of 1:1, and left in culture for 7 days in cIMDM with 5 U/mL IL-2, with a media change at day 4. Viral production was assessed in the supernatant by p24 ELISA (ABL_inc_, Rockville, MA, USA), and percentage of viral inhibition of the different conditions was compared to inhibition from untreated NK cells. To assess the impact of LRAs on cell population proportion, we performed FACS analysis of the cells at the end of the viral inhibition assay. Cells were harvested and surface stained with CD3-PerCP, CD4-FITC, and CD56-PE (BD). The proportion of CD4^+^ T cells was evaluated in the CD3^+^ population, while the proportion of NK cells was evaluated in the whole sample. For the blocking experiments, NKG2D blockade was performed incubating PROST-treated NK cells with pure NKG2D (Miltenty Biotec) during 30 min at room temperature before starting the viral inhibition culture. Success of blockade was checked by flow cytometry.

### Toxicity Assay

Natural killer cells were cultured in cIMDM in the presence or absence of the individual LRAs from our panel for 24 h. After washing, cells were re-suspended in Annexin binding buffer and stained with Annexin V-FITC and 7-AAD (Biolegend, San Diego, CA, USA) following manufacturers’ protocol. Samples were analyzed on the Attune Focusing Cytometer (Applied Biosystems), and the percentage of double-positive cells for both Annexin V and 7-AAD was considered as the non-viable population.

### Cytotoxicity, IFN-γ Production, and Non-Specific Activation Assays

Natural killer cytotoxicity and IFN-γ production were analyzed in co-cultures of primary NK cells and K562 cells (an NK-sensitive target cell line that lacks MHC-I molecules) with and without previous exposure of the NK cells to individual LRAs from our panel. Cytotoxicity was assessed by analyzing the expression of the degranulation marker CD107a, a reliable marker of NK cell cytotoxic activity (28). A total of 100,000 NK cells were co-cultured with the same number of K562 target cells in 96-well plates for 4–6 h in the presence of PE/Cy7-CD107a antibody, clone H4A3 (BD), adding 1 μL of GolgiStop (BD) after the first hour of culture. Cells were then harvested, washed, and surface-stained with CD56-FITC, clone NCAM 16 (BD) in staining buffer for 20 min on ice in the dark. Cells were then fixed with Fixation buffer (Biolegend, San Diego, CA, USA) during 20 min at room temperature in the dark, washed with Perm/Wash buffer twice and intracellularly stained with IFNγ-PE (Biolegend, San Diego, CA, USA) for 20 min. After washing, cells were re-suspended in staining buffer and analyzed in the Attune Focusing Cytometer (Applied Biosystems). To analyze whether NK cells were non-specifically activated by LRA, NK cells were also incubated in the absence of target cells, and CD69 (CD69-PE, clone FN50, from BD) and CD107a expression was analyzed as described.

### Expression of Activating Receptors in NK Cells

A panel of NK cell activating receptors was analyzed by flow cytometry comparing untreated NK cells and cells exposed to individual LRAs from our panel. The following surface monoclonal antibodies were used: CD56-APC/Cy7 (clone HCD56), CD16-Pacific Blue (clone 3G8), NKG2D-Brilliant Violet 510 (clone 1D11), NKp30-PE (clone P30-15), NKp44-AlexaFluor 647 (clone P44-8), NKp46-PE/Cy7 (clone 9E2), and DNAM-1-FITC (clone 11A8) (all from Biolegend, San Diego, CA, USA). Samples were analyzed on LSR Fortessa (Becton Dickinson) cytometer. The expression of each of the receptors was analyzed on the CD56^+^ population using FlowJo X software (Ashland, OR, USA). To set the gates, fluorescence minus one (FMO) controls were used for each individual experiment.

### Expression of NK Ligands on Resting CD4^+^ T Cells

Primary resting CD4^+^ T cells were isolated from healthy HIV-uninfected donors and cultured *in vitro* with individual LRAs from our panel. After 24 h in culture with the LRAs, cells were washed and stained with antibodies against seven different NK ligands. Antibodies used included NTB-A (clone NT-7, Biolegend), HLA-E (clone 3D12, Biolegend), Bw4 (clone REA274, Miltenyi Biotec), CD155 (clone TX24, Biolegend), ULBP-1 (clone 170818, R&R Systems), ULBPB-2 (clone 16590, R&R Systems), and CD48 (clone TU145, BD Pharmingen). Flow cytometry was performed to analyze for changes in cell surface expression. Median intensity of fluorescence was compared to medium/carrier solvent alone (negative control, dimethyl sulfoxide).

### Statistical Analysis

Statistical analyses were performed using GraphPad Prism version 6.07 (GraphPad Software, La Jolla, CA, USA). Data are presented either as raw values or fold change (FC) normalized to untreated NK cells for each of the experiments. Statistical significance was determined with a Wilcoxon matched-pairs signed rank test.

## Results

### NK Cell Antiviral Activity Improves after Prostratin Exposure but It Is Impaired after Panobinostat and Ingenol Treatment

**Figure 1:**
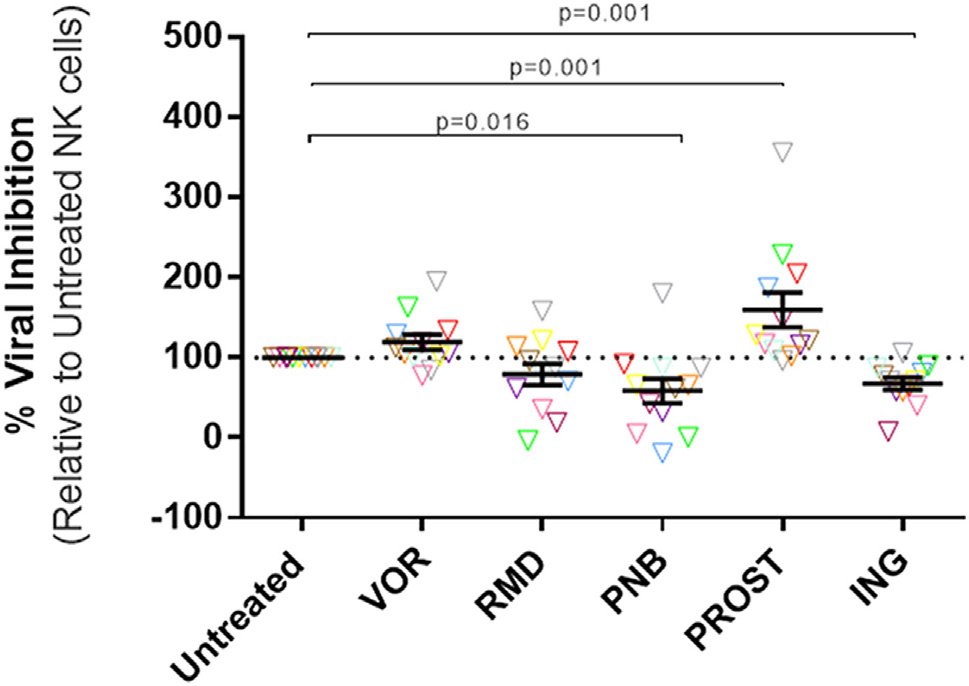
**Antiviral activity of NK cells**. Percentage HIV replication inhibition after 7 days of culture, normalized to untreated NK cells. CD4^+^ T cells were isolated, stimulated, and infected with JR-CSF. Infected targets were cultured with autologous NK cells in triplicate at a ratio 1:1. Each color represents cells from a different donor. At physiologically relevant doses, VOR and RMD did not have significant impact in the antiviral activity of the NK cells, while exposure to PNB and ING impaired NK cell viral inhibition capacity. On the contrary, NK treatment with PROST improves antiviral activity of NK cells. *p*-values were calculated using a Wilcoxon matched-pairs signed rank test. *N* = 12. VOR, vorinostat; RMD, romidepsin; PNB, panobinostat; PROST, prostratin; ING, ingenol.

Viral inhibition capacity of NK cells was tested in autologous cell systems, using NK and CD4^+^ T cells from the same donor. The average purity of NK cells after isolation – measured as the proportion of CD3^−^CD56^+^ – was of 91.26% (SEM = 1.5), and the purity of CD4 cells – measured as the proportion of CD3^+^CD4^+^ – was 93.22 (SEM = 1.6). Results obtained from LRA-treated NK cell conditions were normalized to viral inhibition observed in the untreated NK cell condition. Exposure of NK cells to VOR did not impact the capacity of NK cells to reduce viral replication. However, NK treatment with RMD, PNB, and ING reduced the percentage of viral inhibition (RMD: 79.22%, *p* = 0.176; PNB: 58.6%, *p* = 0.016; ING: 67.69%, *p* = 0.001). Interestingly, PROST exposure improved antiviral activity of NK cells (159.5%, *p* = 0.002) (Figure 1, Figure S1 in Supplementary Material).

To elucidate the mechanisms by which some drugs altered NK cell antiviral activity, we investigated the direct impact of exposure to such drugs on NK cytotoxicity, IFN-γ production, viability, activation, and receptor expression. In addition, at the end of the viral inhibition assay, the cells present in the cultures were stained for NK cell markers (CD56) and T cell markers (CD3 and CD4). Interestingly, we found that proportion of NK cells increased following PROST exposure (153%, SEM = 14.0, *p* = 0.03), while the frequency of CD4 cells within the CD3^+^ population was diminished in the PROST-treated NK group compared to the untreated and all the other conditions (88.1%, SEM 2.7, *p* = 0.03). The increased frequency of NK cells in the culture with PROST was primarily due to an increase in the CD56^bright^ population (216.7%, SEM 28.1, *p* = 0.03). For three donors, we also analyzed absolute numbers of cells, finding that PROST treatment increased cell number, specifically NK number (Figure S2 in Supplementary Material). Finally, we performed a three viral inhibition assays blocking the activating receptor NKG2D, showing that blockade of NKG2D in PROST-treated NK cells caused a decrease in viral inhibition capacity (average of 17.43% viral inhibition after blocking of NKG2D compared to 36.12% without blocking, Figure S3 in Supplementary Material).

### Romidepsin and Panobinostat Are Toxic to NK Cells

**Figure 2:**
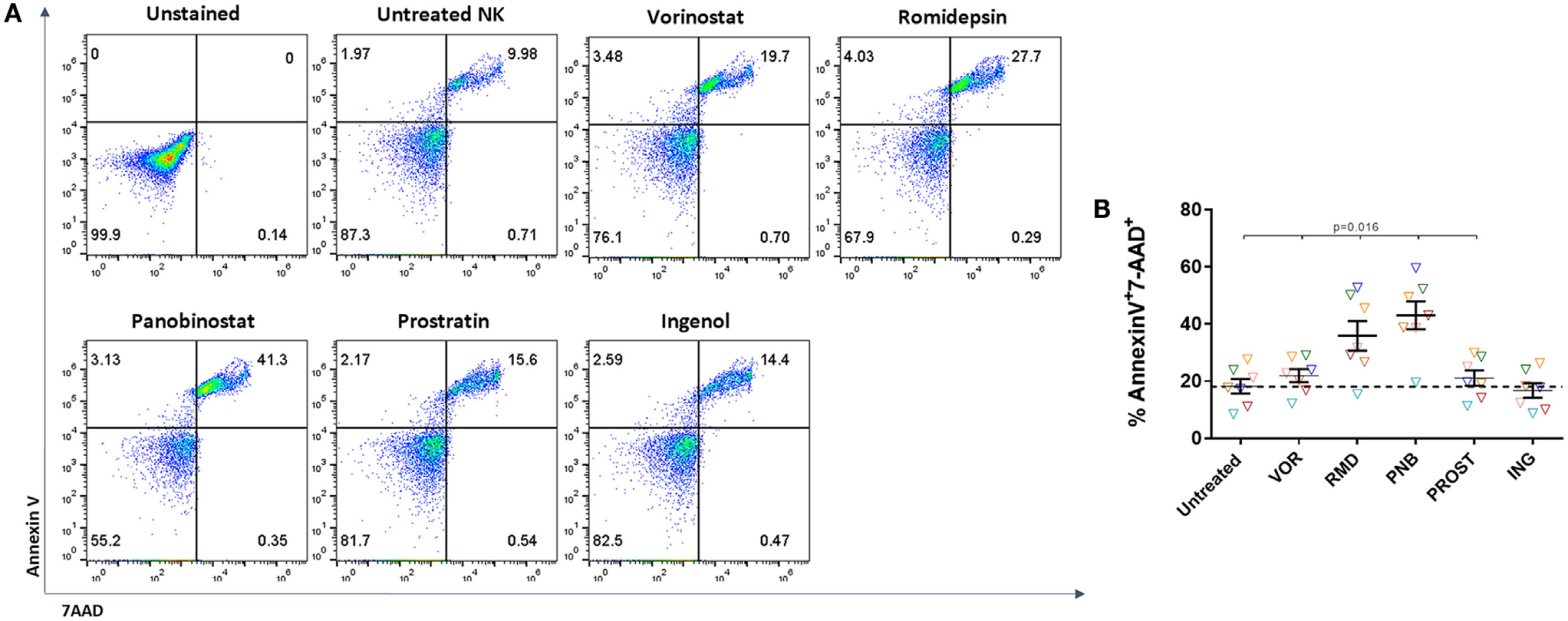
**Toxicity of LRAs on NK cells**. Proportion of dead NK cells after 24-h exposure to the different LRAs. **(A)** Representative FACS analysis showing the gating strategy to identify dead cells according to Annexin V and 7-AAD staining. Cell death was considered when cell were double positive for Annexin V and 7-AAD. **(B)** Proportion of dead NK cells after exposure to the different LRAs, expressed as fold change relative to the untreated condition. Each color represents cells from a different donor. There was a general trend toward a decrease in viability after exposure with all drugs except for ING, but RMD and PNB impact was more pronounced and significantly higher than VOR, PROST, or ING (*p* < 0.02). *N* = 7.

Isolated NK cells were exposed to LRAs for 24 h, washed and stained with Annexin V-FITC and 7-AAD. Dead cells were identified as double-positive cells for Annexin V and 7-AAD (Figure 2A). We observed a general trend toward a decrease in viability after exposure to all drugs (*p* = 0.02) except for ING (*p* = 0.53), but the impact of RMD and PNB was more pronounced, with a mean FC of 2 and 2.5 for RMD and PNB, respectively, compared to VOR and PROST (mean FC = 1.3 and 1.2, respectively) (Figure 2B). In addition, exposure to increasing concentrations of RMD and PNB decreased cell viability in a dose-dependent manner, while viability of NK cells was not reduced following exposure to higher concentrations of VOR, PROST, or ING (Figure S4 in Supplementary Material). As the observed dead cell numbers were high, we compared the viability of untreated NK cells right after isolation and after 24 h in culture. We observed an increase of the apoptotic population (measured as Annexin V+) from 14.8 to 32%. This high rate of cellular death may be due to the lack of IL-15 or IL-2 in the culture.

### LRA Impact on NK Cell-Mediated Cytotoxicity

**Figure 3:**
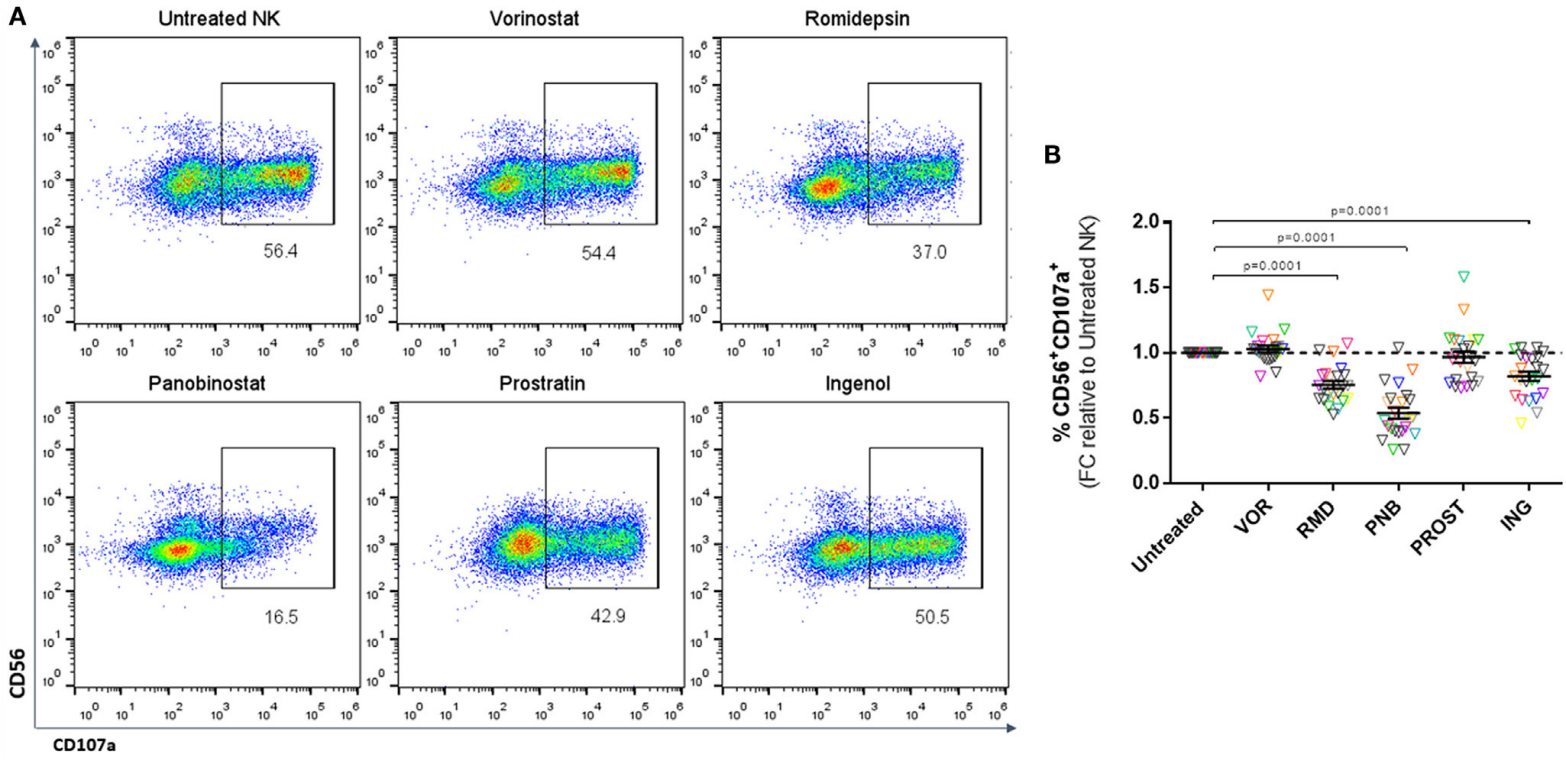
**Cytotoxicity of NK cells**. Proportion of CD56^+^CD107a^+^ cells after 4–6 h culture with K562 cells, expressed as fold change (FC) relative to untreated NK cells. **(A)** Representative FACS analysis plots showing the gating strategy. **(B)** Proportion of CD56^+^ cells expressing the degranulation marker CD107a. Each color represents cells from a different donor. Exposure to PNB, RMD and ING significantly impaired NK degranulation. *N* = 23.

Natural killer cytotoxicity was measured by analyzing the expression of CD107a after co-culture with K562 cells (Figure 3A). Exposure to a physiologically relevant concentration of RMD, PNB, and ING impaired the cytotoxic capacity of NK cells, as shown by a significant decrease in the proportion of CD56^+^CD107a^+^ cells (*p* < 0.0001). However, VOR and PROST did not have a significant impact on NK cytotoxic function (Figure 3B). The observed impairment in NK cell cytotoxic function caused by RMD, PNB, and ING was not due to a direct effect on the viability of NK cells, as we checked in cytotoxic function assays, including a viability stain (Annexin V-FITC and 7-AAD, *n* = 4 donors; data not shown).

We further analyzed whether the impairment in cytotoxic activity was dose dependent. NK cell cytotoxicity showed a dose-dependent reduction after exposure to RMD and PNB, and to some extent to VOR. Interestingly, a higher dose (1000 nM) of ING showed a slight improvement in NK cytotoxic function compared to 100 nM (Figure S5 in Supplementary Material).

### Panobinostat and Prostratin Impair IFN-γ Secretion

**Figure 4:**
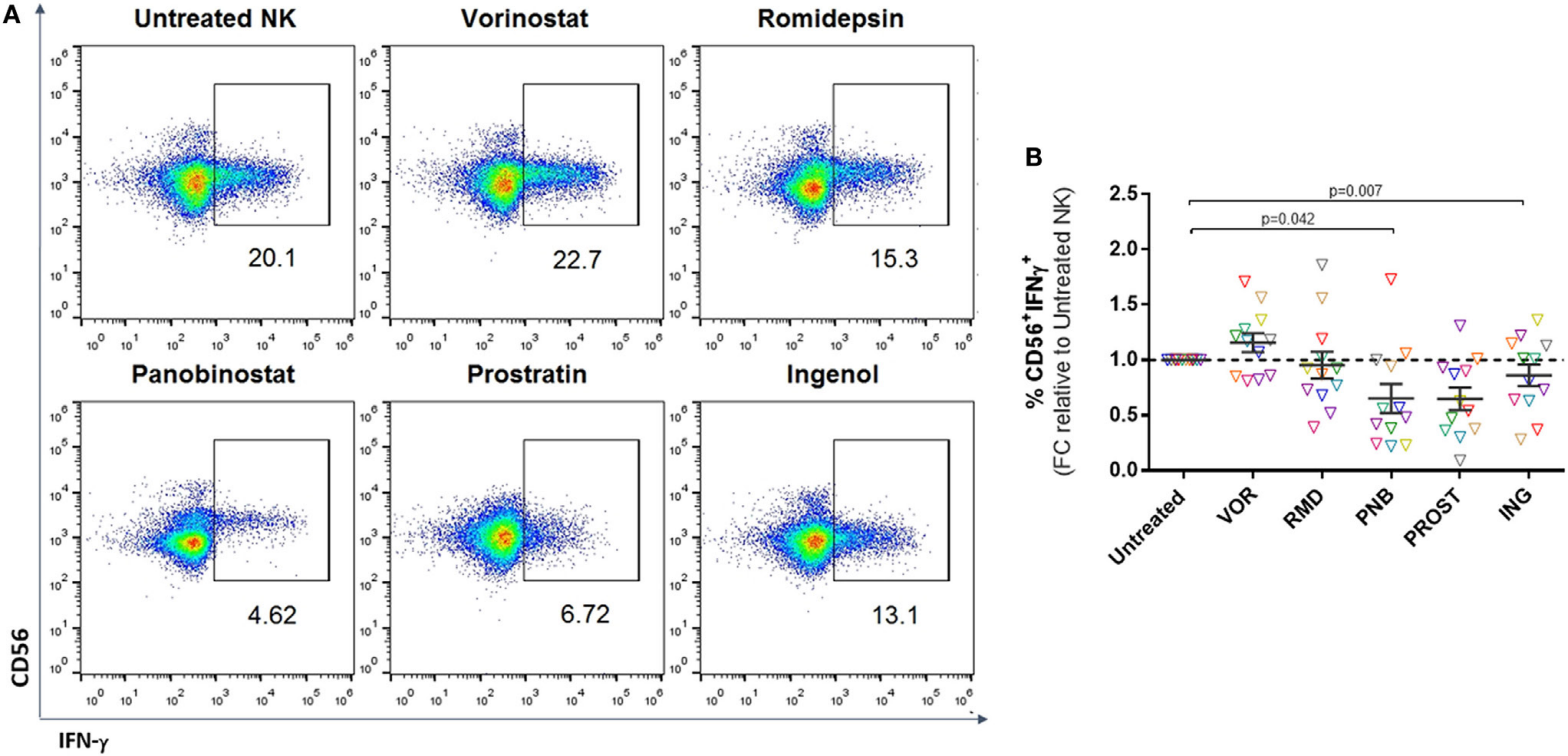
**Cytokine production**. Proportion of CD56^+^IFN-γ^+^ cells after 4–6 h culture with K562 cells. **(A)** Representative FACS analysis showing gating strategy after intracellular staining with IFN-γ. **(B)** Proportion of CD56^+^ cells positive for IFN-γ expressed as fold change relative to the untreated NK condition. Each color represents cells from a different donor. VOR, RMD, or ING do not have a significant effect on IFN-γ production, while PNB and PROST impaired cytokine production. *N* = 12.

Interferon-gamma secretion is an antiviral mechanism employed by NK cells and leads to recruitment and modulation of the activity of other effector cells, including CD8^+^ T cells (29). We analyzed IFN-γ production after a 4–6 h culture with K562 target cells (Figure 4A). FACS analysis after intracellular IFN-γ staining demonstrated that treatment with VOR modestly increased the percentage of NK cells that produced IFN-γ compared to untreated NK cells; however, this did not reach statistical significance. On the contrary, treatment with PNB or PROST resulted in a significant reduction in the number of IFN-γ^+^ NK cells (*p* = 0.05 and *p* = 0.01, respectively) (Figure 4B).

### PKC Agonists Cause Non-Specific NK Cell Activation

**Figure 5:**
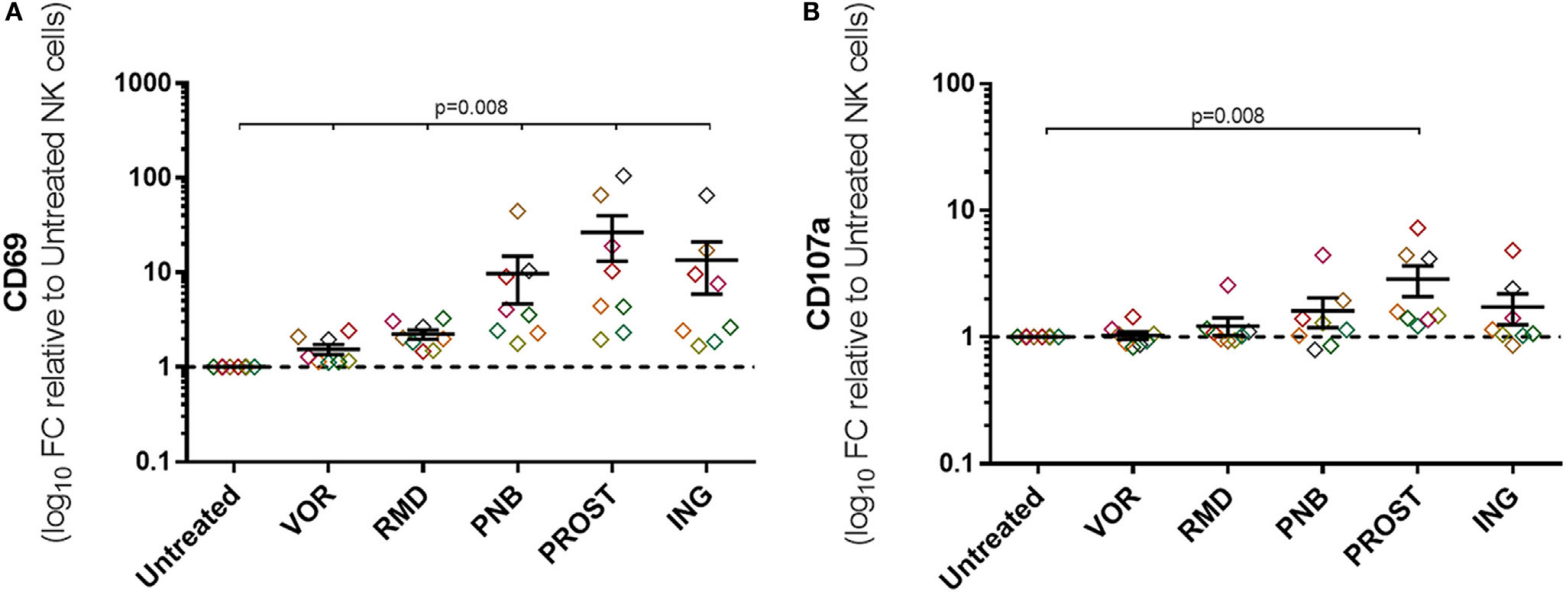
**Non-specific activation of NK cells**. Expression of activation markers on NK cells in the absence of target cells or any other stimuli. Graphs represent log_10_ of the fold change relative to untreated NKs and each color represents cells from a different donor. **(A)** Expression of CD69. **(B)** Expression of CD107a. Exposure to all drugs caused some unspecific activation as measured by CD69 expression, but exposure to the PKC agonists were the most remarkable. PROST also caused a significant increase in CD107a expression. *N* = 8.

To measure non-specific NK cell activation caused by exposure to LRAs, expression of CD69, CD107a, and IFN-γ was measured in the absence of target cells or any other stimulus. Exposure to all drugs caused non-specific activation as measured by CD69 expression (*p* = 0.01), although activation was much greater after treatment with PROST and ING (27- and 14-fold, respectively). Among the HDACi tested, PNB led to an increase in CD69 expression of nearly 10-fold, while VOR and RMD only produced 1.5- and 2.2-fold increase. In addition, exposure to the PKC agonist PROST caused marked increase in CD107a, while the increase caused by ING was more variable (*p* = 0.01 and *p* = 0.06, respectively; Figure 5). IFN-γ production in the absence of target cells was also assessed, and no change was observed after exposure to any of the LRA (data not shown). Figure S6 in Supplementary Material show these results as raw data.

### Expression of Activating Receptors on NK Cells Is Downregulated by Exposure to PNB

**Figure 6:**
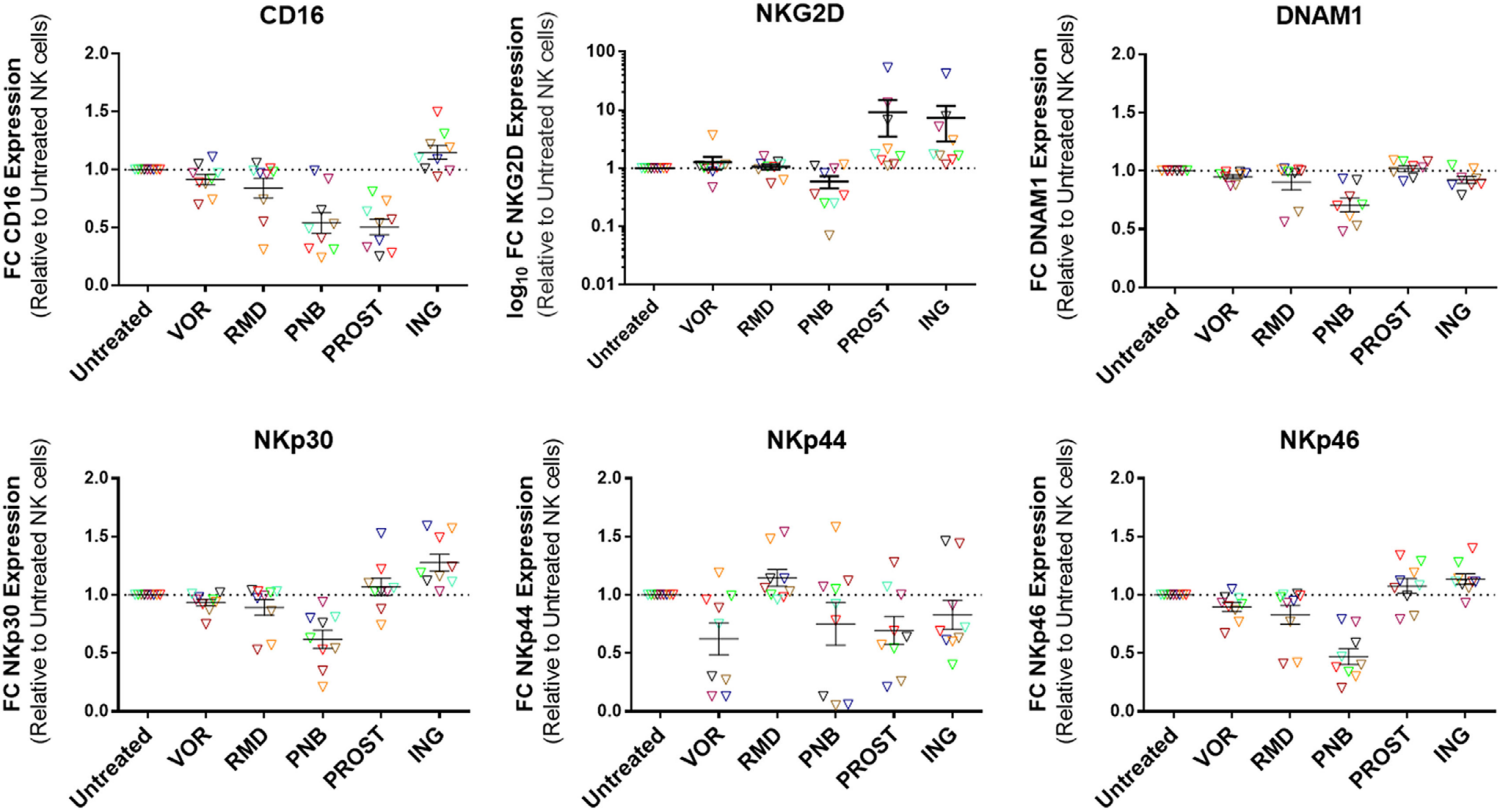
**Expression of activating receptors on NK cells**. Results are expressed as fold change relative to untreated NK cells and each color represents cells from a different donor. PNB caused a general decrease in the expression of activating receptors, as opposed to ING, which caused a significant increase in the expression of all receptors except DNAM1 and NKp44. Of interest, PROST produced a significant increase in the expression of NKG2D. *N* = 9.

Natural killer cells express a wide variety of receptors that enable them to differentiate infected or tumor cells from healthy cells. These include inhibitory, activating, adhesion, and cytokine receptors. The balance of these signals determines whether the NK cell becomes activated or not (30). For this study, we analyzed the expression of activating receptors that have been identified to be important for antiviral activity of NK cells. These included CD16, NKG2D, DNAM-1, NKp30, NKp44, and NKp46 (31). Their expression was measured by flow cytometry after gating on the CD56^+^ population (Figure 6). PNB exposure caused a significant decrease in the expression of all receptors except from NKp44: CD16 (*p* = 0.05), NKG2D (*p* = 0.05), DNAM-1 (*p* = 0.01), NKp30 (*p* = 0.01), and NKp46 (*p* = 0.01). On the contrary, exposure to ING increased the expression of most of the receptors: CD16 (*p* = 0.05), NKG2D (*p* = 0.005), NKp30 (*p* = 0.005), and NKp46 (*p* = 0.01). PROST also caused a significant increase in NKG2D expression (*p* = 0.005), but a decrease in CD16 (*p* = 0.005). Figure S7 in Supplementary Material shows a representative example of the expression of the activating receptors in the presence of these agents.

### Impact of LRAs on NK Ligand Expression in Resting CD4^+^ T Cells

**Figure 7:**
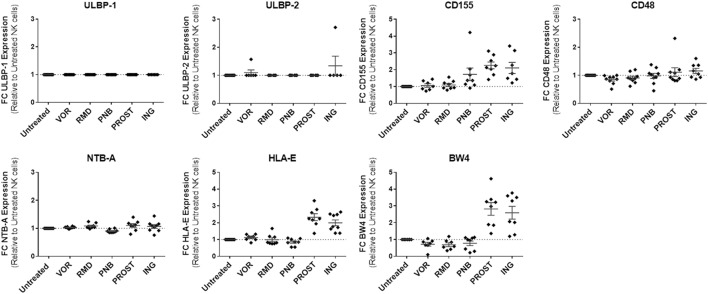
**NK ligand expression on resting CD4^+^ T cells in the presence of LRAs**. LRAs had little effect on NK ligand expression with the exception of PKC agonists, which caused increased expression of the inhibitory NK ligands HLA-E and HLA-Bw4 as well as activating ligand CD155. Median fluorescence intensity was compared to the medium/carrier solvent alone condition (negative control, dimethyl sulfoxide). *N* = 8.

We analyzed by flow cytometry the expression of some ligands on target cells that either trigger NK activation or initiate inhibitory NK signaling by binding to NK inhibitory receptors. The activating ligands included NTB-A, which binds NTB-A on the NK cell surface and leads to activation and secretion of interferon-γ, CD48, which binds 2B4, ULBP-1 and -2, ligands that bind NKG2D and that have been demonstrated to be upregulated in the setting of HIV-1 infection by the viral protein *vpr* (32), and CD155, a NK ligand that binds DNAM-1 on NK cells and induces activation (33). The inhibitory included HLA-E and HLA-Bw4, NK ligands that initiate inhibitory NK signaling by binding to NK receptors NKG2A and KIR3DL1, respectively. The expression of most activating ligands on resting CD4^+^ T cells were unaffected by the LRAs tested, with the exception of CD155, which increased its expression in the presence of both PKC agonists [2.3-fold increase with PROST (*p* = 0.01) and 2.1-fold increase with ING (*p* = 0.02)], as well as PNB (1.7-fold increase, *p* = 0.02). Regarding inhibitory ligands, PKC agonists significantly upregulated expression of HLA-E (ING: twofold, *p* = 0.005; PROST: 2.3-fold, *p* = 0.01) and Bw4 (ING: 2.6-fold, *p* = 0.01, PROST: 2.8-fold, *p* = 0.01). None of the HDACi significantly affected HLA-E expression, although modest trends for decreased HLA-Bw4 expression were observed with all HDACi, reaching statistical significance for VOR (*p* = 0.05). In sum, exposure to PKC agonists PROST and ING significantly increased two inhibitory NK ligands (HLA-E and HLA-Bw4) and one activating ligand (CD155) on the cell surface of primary CD4^+^ T cells, while HDACi exposure had little effect on NK ligand expression on resting CD4^+^ T cells (Figure 7).

## Discussion

For successful implementation of the HIV eradication strategy of latency reversal and clearance, LRAs doses and administration regimens must be selected so that they induce viral antigen expression in latently infected cells but do not interfere with the clearance function of immune effector cells. In this study, we have evaluated the impact of five LRAs on NK cell function. Overall, we observed a heterogeneous effect of the different LRAs evaluated, differing even within each drug class. VOR did not have any significant effect on any of the parameters of NK cell function or viability, while exposure to the other HDACi tested, RMD and PNB, had deleterious effects. Particularly, PNB caused a significant decrease in cytotoxicity, antiviral activity, activating receptor expression, and cytokine secretion of NK cells, along with a decrease in viability. A similar trend was observed for RMD, but milder that the observed with PNB. PKC agonists induced expression of some markers of activation in NK cells and, remarkably, PROST exposure lead to an improvement in antiviral activity.

To our knowledge, no studies of the impact of PNB or PKC agonists on NK cell function have been performed. A previous study reported VOR suppression of NK cell cytolytic activity by impairing granule exocytosis and decreasing expression of activating receptors (34). However, a 96-h incubation with the drug was carried out in those experiments, which exceeds the *in vivo* pharmacokinetic exposure to VOR, cleared in less than 6 h (35). On the contrary, we did not observe any negative impact of VOR on NK cell function. Clinical treatment with RMD in patients with cutaneous T-cell lymphoma induced a decrease of NK cell cytolytic activity, similar to our *ex vivo* results, although interestingly the activity was restored after stimulation with a toll-like receptor agonist (36).

Here, we observed that PNB exposure caused a decrease in the antiviral activity of NK cells. This impairment in viral inhibition was likely due to the reduction in cytotoxicity and IFN-γ production that we also observed, as well as the down-modulation of important activating receptors, such as NKG2D, on the surface of the NK cells. In addition, PNB decreased NK cell viability, so at least part of the decreased antiviral activity observed in the viral inhibition experiments could be due to an actual reduction of number of NK cells in culture, with a consequent reduction in E:T cell ratio. In fact, using our whole dataset, we found that both cytotoxicity and cell viability correlated significantly with a decrease in antiviral activity (Figure S8 in Supplementary Material). PNB has been shown in a study to be the most potent of all HDAC inhibitors (37), and could represent a very promising option for HIV reactivation. However, our *in vitro* results point out the potential deleterious effects that PNB can have on NK cell function, and this should be monitored in future clinical studies. Interestingly, the recent *in vivo* pilot clinical trial with PNB observed that patients with more pronounced proviral DNA decline during PNB treatment had a higher frequency of NK cells (22), suggesting that the function of the innate immune system should be monitored during latency reversal and clearance studies.

On the other hand, we observed an improvement in NK antiviral activity after exposure to PROST. This observation could be due to the increase in NK activation upon culture with the drug; however, it is also possible that PROST remained attached to cellular membranes despite washing after drug exposure as has previously been suggested (38), leading to blunted HIV infection in target cells. When we analyzed the cell composition of the cultures after a 7-day viral inhibition assay, we consistently observed that in the cultures where NK cells were exposed to PROST, the CD3^−^CD56^+^ subset was increased compared to all the other conditions, with an especially marked increase in the CD56^bright^ population, while the proportion of cells expressing CD4 within the CD3^+^ population was decreased. Moreover, exposure to PROST upregulated the expression of NKG2D, which is a NK cell activating receptor known for its importance in NK cell antiviral activity (39, 40). Thus, the increase in antiviral activity observed after PROST exposure was most likely due to a combination of several effects, including changes in both the target population and in the effector population. This may have interesting implications for *in vivo* HIV-1 eradication strategies in which PKC agonists might simultaneously induce several desirable effects: reactivation of latently infected cells, inhibition of viral replication, and enhancement of the antiviral effect of NK cells. ING, the other PKC agonist tested in our experiments, did not have such a marked effect in any of the functional characteristics analyzed, but we did observe upregulation of NKG2D and some activation measured by CD69. Beyond the specific characteristics of each of these components of the PKC agonist family, a possible reason for the difference in the magnitude of the effect observed with PROST and ING could be the dose of each drug used in our experiments. In fact, when a higher dose (1 μM) of ING was used for the viral inhibition experiments, an improvement in NK cell antiviral activity was observed (mean of twofold compared to untreated NK cells, Figure S9 in Supplementary Material). However, we also observed a decrease in IFN-γ production when NK cells were exposed to PKC agonists. This conflicts with our observations of the increase of the CD56^bright^ population, given that generally CD56^dim^ subpopulations are more cytotoxic, while CD56^bright^ are able to produce IFN-γ (41). We also observed a decrease in CD16 expression on NK cells treated with PROST, which in this case would correlate with the increase in the CD56^bright^ subpopulation, as CD16 is expressed largely in CD56^dim^ subsets. Down-modulation of CD16 is concerning given that ADCC is mediated by antibody engagement to this receptor, and further study is required to determine whether this decrease in expression has functional consequences.

Our experiments were performed using unstimulated NK cells, with the aim of recapitulating *in vivo* exposure *to* LRAs. However, for immunotherapeutic purposes, an alternative strategy involves *ex vivo* stimulation and expansion of NK cells, with the intention of improving their cytotoxic potential. Susceptibility of these expanded cells to LRAs may differ from what we have observed in non-stimulated cells, as it has been already shown for impact on CTL (17). In fact, we observed a reduction in K562 target cell lysis when expanded NK cells were exposed to PROST (data not shown). On the contrary, and although we did not observe relevant impact of VOR on NK cell function, Schmudde et al. reported an impairment of NK cell degranulation by VOR exposure when cells were not stimulated, but no impact if NK cells were previously stimulated with IL-2 (42).

In addition to investigating the direct impact of LRAs on NK cells, we analyzed the impact of exposure to these agents on target cells relevant for HIV infection (resting CD4^+^ T cells) to assess expression of surface ligands that would render them more susceptible to NK cell recognition and clearance. Overall, we did not observe a striking effect on ligand expression on CD4^+^ T cells, with the exception of a modest increase in HLA-E and BW4 (inhibitory ligands), and CD155 (an activating ligand) when cells were exposed to PKC agonists. Different observations have been reported from oncology studies, where it has been consistently reported that HDACi upregulate the expression of NKG2D ligands on tumor cells (43–46), helping their recognition by innate immune system. On the other hand, it has also been seen that HDACi can down-modulate the expression of NKp30 ligands on tumor cells, reducing NKp30-dependent effector functions of NK cells (47).

To achieve clinically significant reversal of HIV-1 latency, several studies suggest that combinations of mechanistically different LRA will be needed (48–50). Our results add an additional factor to consider when designing an adequate LRA combination, as not only reactivation potency should be taken into account but also the impact that each of the drugs have on immune effector function. Thus, if a certain LRA is selected to be used because of its potency for reactivating the reservoir but it has shown to cause an impair in immune function, the second component of the LRA combination ideally should have proven to cause an improvement to some extent in the effector activity. The optimum situation for cure strategies would be finding compounds that simultaneously can disrupt latency and boost the immune response, and some agents with these capabilities are beginning to be described (51).

In summary, we have evaluated *ex vivo* the impact of five different latency-reversing agents on the effector function, phenotype, and viability of NK cells. This is of clinical relevance given the necessity of a potent immune response after reactivation of the latent HIV reservoir in order to achieve viral eradication. We have found a heterogeneous effect of the different agents studied, highlighting the lack of impact of VOR, the negative effects of PNB and RMD, and the potential beneficial impact of PKC agonists. Impact of LRA on immune function should be considered when designing LRA combinations to reactivate the latent HIV-1 reservoir. This is the first study to address the impact of LRAs on innate immune functions in the context of HIV-1 eradication, and demonstrate the importance of further evaluation of NK cell function. However, *in vitro* results might differ from *in vivo* effects. Our analysis of the effect of LRAs on NK cell function is, of necessity, only a preliminary one. In clinical trials, multiple doses of LRAs will be given over time, a phenomenon that is more difficult to model. Thus, innate immune function should be evaluated in HIV-1 positive patients undergoing latency reversing therapy.

## Author Contributions

CG and DM conceived the study. AS performed the experiments for ligand expression on target cells, MC performed target lysis experiments, and CG conducted all other experimental work. NS-S, AS, JK, EB, and VP contributed with ideas and experimental design of the study. CG wrote the manuscript, with edits from DM, NS-S, AS, MC, and VP. All authors revised the contents of the article.

## Conflict of Interest Statement

The authors declare that the study was conducted in the absence of any commercial or financial relationships that could constitute a potential conflict of interest.

## Abbreviations

IFN-γ, interferon-gamma; ING, ingenol; LRA, latency reversing agents; PNB, panobinostat; PROST, prostratin; RMD, romidepsin; VOR, vorinostat.
