# Supplementary material for: HIV Latency-Reversing Agents Have Diverse Effects on Natural Killer Cell Function
Source: Front Immunol. 2016 Sep 21;7:356. doi: 10.3389/fimmu.2016.00356 (PMC5030263; doi:10.3389/fimmu.2016.00356)
Supplement: Supplementary file 1 [file Data_Sheet_1.docx]

**Supplementary figure S1.** **Antiviral activity of NK cells.** HIV p24 *gag* antigen production after 7 days of culture. CD4^+^T cells were isolated, stimulated and infected with JR-CSF. Infected targets were cultured with autologous NK cells in triplicate at a ratio 1:1. Each color represents cells from a different donor. p values were calculated using the Wilcoxon matched-pairs signed rank test. N=12. VOR, vorinostat; RMD, romidepsin; PNB, panobinostat; PROST, prostratin; ING, ingenol.

**C.**

**B.**

**A.**

**D.**

**Supplementary figure S2.** NK and CD4^+^T cell frequency at day 7 of viral inhibition assay. Results are normalized to the untreated NK cell condition. Each color represents cells from a different donor. **A.** Proportion of CD3^-^CD56^+^ NK cells. **B.** Proportion of CD3^-^CD56^bright^ cells. **C.** Proportion of CD4^+^ expressing cells within the CD3^+^ population. p values were calculated with a Wilcoxon matched-pairs signed rank test. n=6. **D.** Absolute number of cells from 3 donors. Bars show the total number of cells, indicating in grey the number of NK cells and in dark grey the number of CD3^-^CD56^bright^.

**Supplementary figure S3.** **Antiviral activity of NK cells is decreased after blocking NKG2D.** HIV p24 *gag* antigen production after 7 days of culture of targets alone, targets with Prostratin treated NK cells or targets with PROST-treated NK cells after blocking the activating receptor NKG2D. p values were calculated with a paired t test. N=3.

**Supplementary figure S4.** Cytotoxic effect of increasing concentrations of latency reversing agents on natural killer cells. Cell death is measured as cells positive for both Annexin V and 7-AAD.

**Supplementary figure S5.** Degranulation activity of natural killer cells after exposure to increasing concentrations of latency reversing agents. Degranulation is measured as proportion of NK cells expressing the degranulation marker CD107a after culture with the target cell line K562.

**Supplementary figure S6. Non-specific activation of NK cells.** Expression of activation markers on NK cells in the absence of target cells or any other stimuli. Graphs represent proportion of NK cells expressing the activation markers, and each color represents cells from a different donor. **(A)** Expression of CD69. **(B)** Expression of CD107a.

**
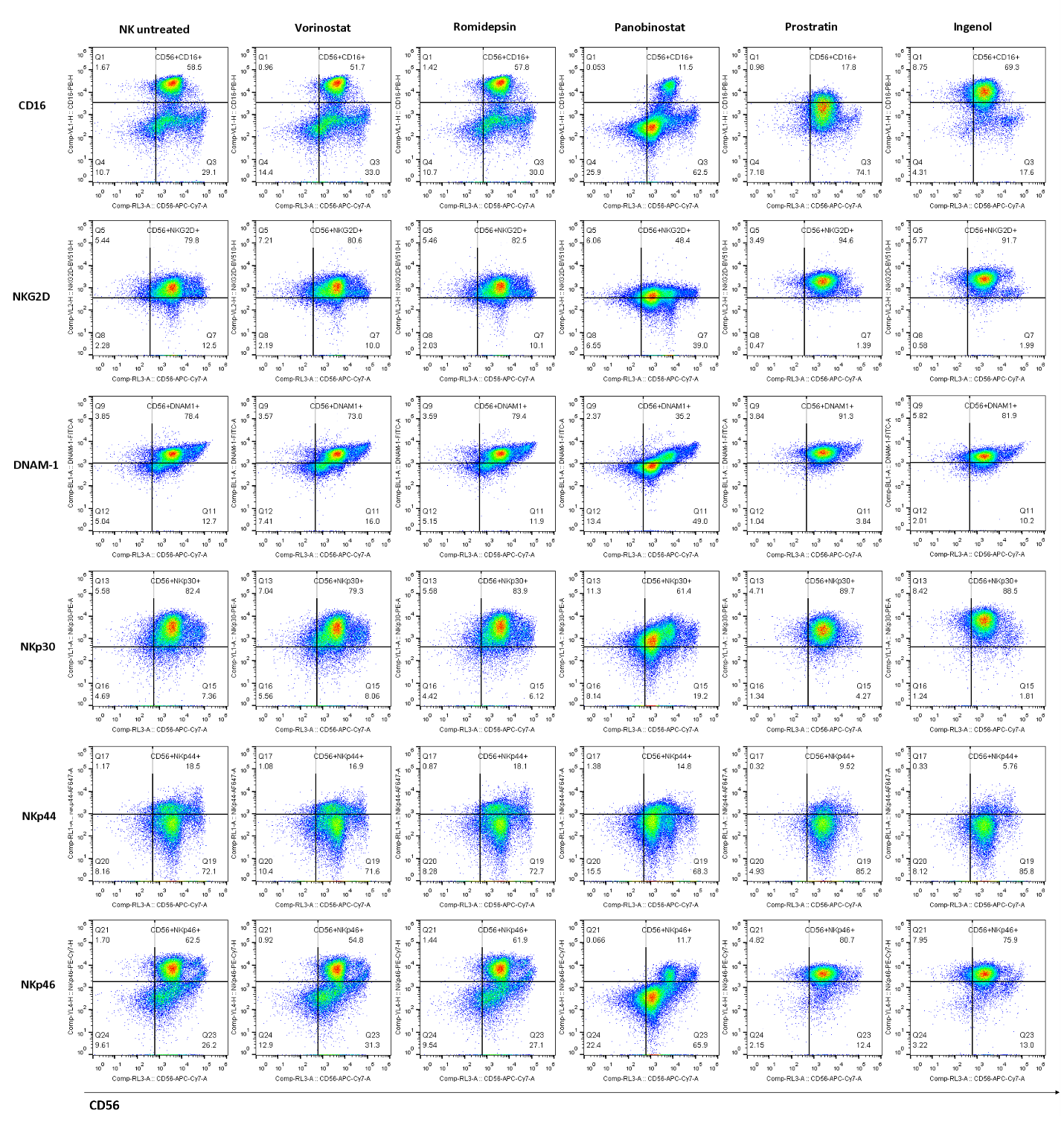
**

**A.**

**B.**

| **Donor 40** | **MFI** | | | | | |
| --- | --- | --- | --- | --- | --- | --- |
|  | **Untreated NK** | **VOR** | **RMD** | **PNB** | **PROST** | **ING** |
| **CD16** | 17580 | 15535 | 18285 | 3021 | 2751 | 12093 |
| **NKG2D** | 1025 | 1418 | 1099 | 565 | 2050 | 2520 |
| **DNAM-1** | 2158 | 2043 | 2243 | 1195 | 2696 | 1794 |
| **NKp30** | 2542 | 2228 | 2742 | 1072 | 2357 | 5792 |
| **NKp44** | 659 | 583 | 624 | 577 | 452 | 390 |
| **NKp46** | 5104 | 4037 | 4959 | 860 | 3793 | 3779 |

**Figure S7. Representative plots of receptor expression analyzed in one donor. A.** Individual NK receptor expression (y-axis) in CD56^+^ cells (x-axis). **B.** MFI values for each receptor analyzed.

C.

B.

A.

**Supplementary figure S8. Linear regression analyses of NK cell degranulation, antiviral activity and cell death after exposure to the different latency reversing agents.** Each dot represent cells from the same patient treated with the same LRA. Degranulation was measured as proportion of NK cells expressing the degranulation marker CD107a after culture with the target cell line K562. Antiviral activity is expressed as viral replication inhibition. Cells are considered dead when they are positive for both Annexin V and 7-AAD. **A.** Correlation of degranulation and toxicity. **B.** Correlation of antiviral activity and toxicity. **C.** Correlation of degranulation and antiviral activity.

**Supplementary figure S9. Effect of ING on NK inhibition of HIV replication.** Reduction of HIV p24 antigen production at 7 days of culture mediated by the addition of NK cells normalized to 100%. Increasing concentrations of ING caused an improvement in NK antiviral activity. N=3
